# Echo-Time and Field Strength Dependence of BOLD Reactivity in Veins and Parenchyma Using Flow-Normalized Hypercapnic Manipulation

**DOI:** 10.1371/journal.pone.0024519

**Published:** 2011-09-06

**Authors:** Christina Triantafyllou, Lawrence L. Wald, Richard D. Hoge

**Affiliations:** 1 A.A. Martinos Imaging Center at McGovern Institute for Brain Research, Massachusetts Institute of Technology, Cambridge, Massachusetts, United States of America; 2 Department of Radiology, Harvard Medical School, A.A. Martinos Center for Biomedical Imaging, Massachusetts General Hospital, Charlestown, Massachusetts, United States of America; 3 Harvard-Massachusetts Institute of Technology (MIT) Division of Health Sciences and Technology, Massachusetts Institute of Technology, Cambridge, Massachusetts, United States of America; 4 Unité de Neuroimagerie Fonctionelle, Centre de recherche de l'institut universitaire de gériatrie de Montréal, Montreal, Canada; 5 Université de Montréal, Montreal, Canada; National Institute of Mental Health, United States of America

## Abstract

While the BOLD (Blood Oxygenation Level Dependent) contrast mechanism has demonstrated excellent sensitivity to neuronal activation, its specificity with regards to differentiating vascular and parenchymal responses has been an area of ongoing concern. By inducing a global increase in Cerebral Blood Flow (CBF), we examined the effect of magnetic field strength and echo-time (TE) on the gradient-echo BOLD response in areas of cortical gray matter and in resolvable veins. In order to define a quantitative index of BOLD reactivity, we measured the percent BOLD response per unit fractional change in global gray matter CBF induced by inhaling carbon dioxide (CO_2_). By normalizing the BOLD response to the underlying CBF change and determining the BOLD response as a function of TE, we calculated the change in R_2_^*^ (ΔR_2_^*^) per unit fractional flow change; the Flow Relaxation Coefficient, (FRC) for 3T and 1.5T in parenchymal and large vein compartments. The FRC in parenchymal voxels was 1.76±0.54 fold higher at 3T than at 1.5T and was 2.96±0.66 and 3.12±0.76 fold higher for veins than parenchyma at 1.5T and 3T respectively, showing a quantitative measure of the increase in specificity to parenchymal sources at 3T compared to 1.5T. Additionally, the results allow optimization of the TE to prioritize either maximum parenchymal BOLD response or maximum parenchymal specificity. Parenchymal signals peaked at TE values of 62.0±11.5 ms and 41.5±7.5 ms for 1.5T and 3T, respectively, while the response in the major veins peaked at shorter TE values; 41.0±6.9 ms and 21.5±1.0 ms for 1.5T and 3T. These experiments showed that at 3T, the BOLD CNR in parenchymal voxels exceeded that of 1.5T by a factor of 1.9±0.4 at the optimal TE for each field.

## Introduction

In recent years there has been an emphasis on the use of increasingly high field strengths in functional MR imaging. While there are undoubtedly benefits to adoption of higher field strength instruments, quantitative data demonstrating their advantages is essential to justify the increased cost and complexity. Conversely, an understanding of the capabilities and limitations of lower field systems such as 1.5T is important to guide appropriate utilization of the large installed base of clinical 1.5T systems.

The overall objective of this paper is the characterization of *sensitivity* and *specificity* of gradient-echo BOLD functional MRI at 1.5T and 3T over a range of echo-times, and to compare their performance at each field strength. We study the sensitivity and specificity of BOLD using a controlled global stimulus (hypercapnia). In particular, we aim to identify the optimal echo-time (TE) at two different magnetic field strengths in areas of cortical gray matter and resolvable veins, in order to maximize specificity to cortex or maximize sensitivity (by avoiding the contribution of the vascular component). Because we compare the BOLD responses at different field strengths and across different scanning sessions, we introduce and define a new quantitative index of BOLD sensitivity, the Flow Relaxation Coefficient (FRC), using flow-responses induced by CO_2_ inhalation (i.e. direct Cerebral Blood Flow measurements) to control for inter-session variations and ensure that the manipulation is equivalent across imaging sessions. Given the broad availability of both 1.5T and 3T systems and the prevalence of gradient-echo BOLD fMRI at these field strengths, we have focused on these field strengths.

Since increases in susceptibility effects that occur with increased field strength affect both the functional responses [1], [2] and physiological noise [3], it is important to determine the net increase in the contrast-to-noise ratio (CNR) that occurs with field strength as it is this ratio that is the ultimate determinant of sensitivity in functional MRI experiments. The CNR increases in, for example, visual stimulation experiments have been compared for different field strengths [1], [2], [4], [5], and have also been examined for motor activation [6]. Poser and Norris [7] have investigated the sensitivity of BOLD imaging at 7T by measuring and combining responses at different echo-times, while Olman et al. [4] have compared the sensitivity of spin-echo BOLD imaging at 3T and 7T.

In addition to *sensitivity*, a second important criterion in functional imaging is *specificity*. In BOLD fMRI the major challenge in achieving specificity is the large amplitude of responses in venous blood vessels that drain activated tissue regions. Venous responses generally exceed parenchymal responses by an appreciable factor [1]. Most of the above literature has promoted the notion that higher fields offer better specificity against macro-venous responses. Spin-echo BOLD responses are also a subject of considerable interest for improving specificity, especially at ultra high field strengths (e.g. 7T and above), [8], [9]. Uludag et al. [5] have described a comprehensive model of susceptibility-based MRI contrast, from micro and macro vasculature, which explains important differences between spin- and gradient-echo acquisitions at different field strengths and echo-times, based on simulations. The present study contributes systematically acquired data to explore the significance of venous responses at 3T and compare against the 1.5T field strength system to investigate further, whether this effect has increased by the field strength or eliminated.

A limitation of using sensory stimuli to characterize BOLD contrast, as done in the above studies, is that there is uncertainty in establishing the “ground truth” of where the activation actually occurs. In this respect, the use of hypercapnia as a reference condition is that, being a global manipulation, there is no need to identify “activated” tissue regions through statistical methods. This avoids some of the circularity that may arise when small, noisy activation signals are characterized by sampling regions localized using statistical methods based on those same signals. In the case of hypercapnia, all parenchymal gray matter and associated veins are “activated” in the sense of undergoing increased blood flow and the precise location of the region of interest analyzed is not important as long as voxels can be categorized into appropriate compartments (vascular, parenchymal). Furthermore, the global activation allows use of larger regions of interest without risk of selection bias. Using conventional task-activated definitions of analysis regions may result in the over-representation of veins due to the fact that veins generally provide the highest CNR activation. With the whole cortex uniformly activated, regions free of large veins can be readily selected to estimate the parenchymal response amplitude.

Previous investigators have used carbon dioxide (CO_2_) inhalation to study BOLD responses in humans. Bandettini *et al* normalized BOLD activation images by maps of CO_2_-induced BOLD signal change in an attempt to attenuate large responses associated with veins [10]. Davis *et al*, Hoge *et al*, and others have used hypercapnic calibration methods to estimate changes in the cerebral metabolic rate of O_2_ consumption [11], [12], [13], and Corfield and collaborators have investigated the additivity of neuronal and global increases in BOLD signal [14]. Other researchers [15] have used hyperoxia to produce BOLD signal increases which can be calibrated by using the change in end-tidal O_2_ to estimate the venous O_2_ saturation. Cohen *et al.* [6] have also used CO_2_-induced ASL signals to normalize BOLD responses to neuronal activation for the purpose of improving comparison of results acquired on different scanning systems.

In addition to the magnetic field strength, the TE of the pulse sequence will also play a role in determining both sensitivity and specificity. Differentiation of the signal equation for a T_2_^*^-weighted acquisition shows that the maximum effect will be observed when the TE is equal to the T_2_^*^ value of the tissue compartment in question. Since the T_2_^*^ value of large veins is considerably shorter than that of gray matter, the choice of TE can be expected to play a role in determining both sensitivity and specificity.

An additional challenge to comparing BOLD responses at different field strengths using flow-responses induced by CO_2_ inhalation is to ensure that the manipulation is equivalent across imaging sessions and systems. Although breathing a fixed concentration of inspired CO_2_ offers advantages as a repeatable reference condition, it is still possible that the actual change in arterial CO_2_ may vary between sessions, since changes in breathing rate will affect the degree of hypercapnia achieved. To control for possible differences in the Cerebral Blood Flow (CBF) response achieved during the sessions on the different scanners, we embedded Arterial Spin-Labeling (ASL) based flow measurements in the relevant BOLD acquisitions. This allowed us to compare BOLD reactivity by using the ratio of the *percent change in BOLD signal per unit of percent change in CBF signal*. This ratio is likely to be a more invariant reflection of the BOLD sensitivity for probing different tissue compartments and field strengths. By calibrating the stimulus using CBF measurements and determining the BOLD response as a function of TE, we calculated the change in R_2_^*^ (ΔR_2_^*^) per unit fractional flow change (Flow Relaxation Coefficient, FRC) for both field strengths (1.5T and 3T) and each component: parenchymal and major veins.

### Theory

Following the notation used by Hoge and colleagues [12], we review the contributions to the percent BOLD response per unit fractional CBF change in response to inhaled CO_2_. The transverse relaxation rate R_2_^*^ is assumed to be the sum of the de-oxyhemoglobin (*dHb*) contribution, R_2_^*^|_dHb_, and a relaxation rate term due to other sources, R_2_^*^|_other_:
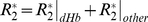
(1)Given that the relationship between R_2_^*^ and Cerebral Blood Volume (*CBV*) can be expressed as:
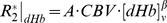
(2)where *A* is dependent on the field strength and the sample under study, [dHb]_v_ is the venous de-oxyhemoglobin concentration and *β* is a constant defined to have values between 1 and 2, also depending on the field strength and venous blood volume fraction within a voxel. The change in the transverse relaxation rate, ΔR_2_^*^|_dHb_ is expressed by:
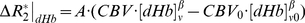
(3)The fractional BOLD signal response as a function of TE can be expressed as:
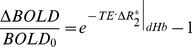
(4)This expression (Eq. 4) can be approximated for small changes using the following linearization:
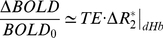
(5)By calibrating the BOLD response using direct CBF measurements, Eq. 5 becomes:
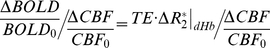
(6)We can then determine the Flow Relaxation Coefficient (FRC) as the change in R_2_^*^|_dHb_ (ΔR_2_^*^|_dHb_) per unit fractional flow change:
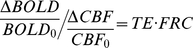
(7)


## Methods

MR Imaging was performed on a Siemens Sonata 1.5T and a Siemens Trio 3T system (Siemens Healthcare, Erlangen, Germany). The same four healthy human subjects (all male, mean age 27±5yrs) were scanned on both imagers using a commercial 8-channel phased array receive head coil and a whole-body transmit coil for excitation and ASL. Written informed consent was obtained from all the subjects for an experimental protocol approved by the institutional review board of the Massachusetts General Hospital. Head immobilization was carried out using foam pads. Automatic slice prescription, based on alignment of localizer scans to a multi-subject atlas, was used to achieve a consistent slice position across scanners and multiple scanning sessions.

### Manipulation of Global Cerebral Blood Flow

During MR Imaging, we induced hypercapnia by administering a CO_2_/air mixture through a non-rebreathing face mask (Hudson RCI Model 1069) worn by each subject. Each scanning run included two intervals of air/CO_2_ inhalation, each of two minutes duration. These periods of hypercapnia were bracketed by two minute intervals during which subjects breathed normal air, for a total duration of ten minutes per run (2 min air/2 min air+CO_2_/2 min air/2 min air+CO_2_/2 min air). The baseline condition was always inhalation of atmospheric composition medical air (CO_2_<300 ppm) delivered at 16 L/min while attending to a neutral visual display. Hypercapnic episodes were initiated during scanning runs by switching the breathing gas to a mixture of CO_2_∶O_2_∶N_2_ at 7%∶21%∶72% respectively (BOC Ltd.) and medical air. Subjects were instructed to breathe at a constant rate, which was easily maintained to within one breath per minute. Pulse rate and arterial oxygen saturation were also monitored (Oxygen/Pulse Monitor, InVivo Inc.) and these remained constant (i.e. changed by less than 2% and pulse rate by less than 5 bpm) throughout hypercapnia experiments. Although end-tidal CO_2_ (ETCO_2_) was not measured in the present study, the manipulation described here has been used in numerous previous studies and found to deliver changes in end-tidal CO_2_ ranging from 5.6 mmHg [16] to 13 mmHg [17]. Since the ETCO_2_ changes elicited by a fixed mixture of inspired gas could be somewhat variable in different subjects, we chose the approach of normalizing BOLD responses by the CBF change elicited by the CO_2_, which is ultimately more relevant as an ‘input’ to the BOLD signal mechanism.

### Data Acquisition

For each subject and each field strength, a total of three scans were performed; two runs of a multi-echo EPI acquisition and one run of ASL acquisition for perfusion imaging. BOLD measurements were performed using a multi-echo gradient-echo EPI sequence. Ten 3 mm thick slices with inter-slice gap = 1.5 mm were positioned parallel to the AC-PC line. The imaging parameters were TR = 3000 ms, 200 time points, FOV = 192×192 mm^2^, matrix = 64×64. Nine echo-times were selected at each field strength to cover a range of the T_2_^*^ decay: 11, 23, 35, 47, 59, 71, 83, 95, 107 ms at 1.5T and 8, 21, 35, 48, 61, 75, 88, 101, 115 ms at 3T. To achieve short echo-time, the images were acquired and reconstructed using the parallel imaging method GRAPPA with acceleration factor of two [18]. The block design paradigm alternated between 2 min periods of baseline and 2 min of global stimulus (breathing CO_2_ mixture) as described above. Within each scanning session, the BOLD protocol was repeated twice for each subject (2 runs). To measure the relative CBF change of each subject during hypercapnia, for later use as a normalizing factor, perfusion weighted imaging was also performed using a PICORE–QUIPPS2 ASL EPI based perfusion sequence [19]. The imaging parameters were kept the same as the ones used in the BOLD experiments, except that the TE was kept constant at 30 ms and 25 ms for 1.5T and 3T respectively (allowing simultaneous extraction of BOLD signals), and the inversion times were PASL-TI1 = 700 ms, PASL-TI2 = 1400 ms. A 15 cm labeling slab was applied with a 1.5 cm gap from the bottom of the first slice. The same paradigm as before was applied during ASL imaging, so that both CBF and BOLD measurements would be available (using the even-numbered control scans for BOLD contrast).

### Data Analysis

The effects of head movement were minimized using motion-correction techniques adapted from AFNI [20]. Linear trends in the image intensity were also removed from the time series at each TE. Pixel-wise T_2_^*^ maps were generated by fitting the signal intensities from the various TEs to a mono-exponential decay model.

Following motion correction, all functional data were spatially smoothed using a 6 mm Gaussian kernel. BOLD response amplitudes were determined from the multi-echo data sets, by fitting a General Linear Model (GLM) plus correlated noise at each echo, using the software package NeuroLens [21]. The model parameters for the hemodynamic response used in GLM fitting were chosen empirically to have delay of 10 sec and width of 30 sec to approximate the CO_2_ response. After convolving with the square-wave block design, this combination of hemodynamic response parameters yielded a regressor with quasi-exponential transition phases that plateaued to the new steady-state in slightly less than one minute, closely approximating the BOLD step response produced by the CO_2_ manipulation [11]. The response magnitudes were calculated from the model parameters (betas) fit in the GLM procedure.

The BOLD signal amplitude was then estimated using a region of interest analysis (ROI). The ROIs were carefully selected within the cortical gray matter to exclude large blood veins (by visual inspection, of the T_2_^*^-weighted EPI time-series, in which veins are readily recognizable as dark structures against the brighter background of gray matter and CSF). Results were averaged over all ROIs on each echo and for each subject. For comparison, the BOLD contrast and the signal intensity were also measured on large (1–2 mm) veins, defined by visual inspection, in the T_2_^*^-weighted EPI scans.

Pixel-wise maps of relative perfusion were calculated from the ASL data. The EPI time series were motion corrected and spatially smoothed with 6 mm Gaussian kernel followed by pair-wise subtraction of the inverted from the non-inverted scans. GLM analysis of the perfusion datasets was performed with same parameters as described above, with an additional analysis of the image sequence derived by extracting the even-numbered control scans, which exhibit pure BOLD contrast. These BOLD measurements were only used to ensure BOLD activations were consistent across the ASL acquisition and the multi-echo runs (data not shown). A single global CBF change, expressed as percent, was then estimated from the generated perfusion maps using ROI analysis (areas of parenchyma) in the whole brain. The blood flow measurements reflect underlying flow responses in the different sessions and were further used to calibrate the BOLD activations by calculating the FRC. This single global flow response was used as the normalization factor to compensate for individual variations in the physiological response to our fixed CO_2_/air mixture.

## Results

**Figure 1:**
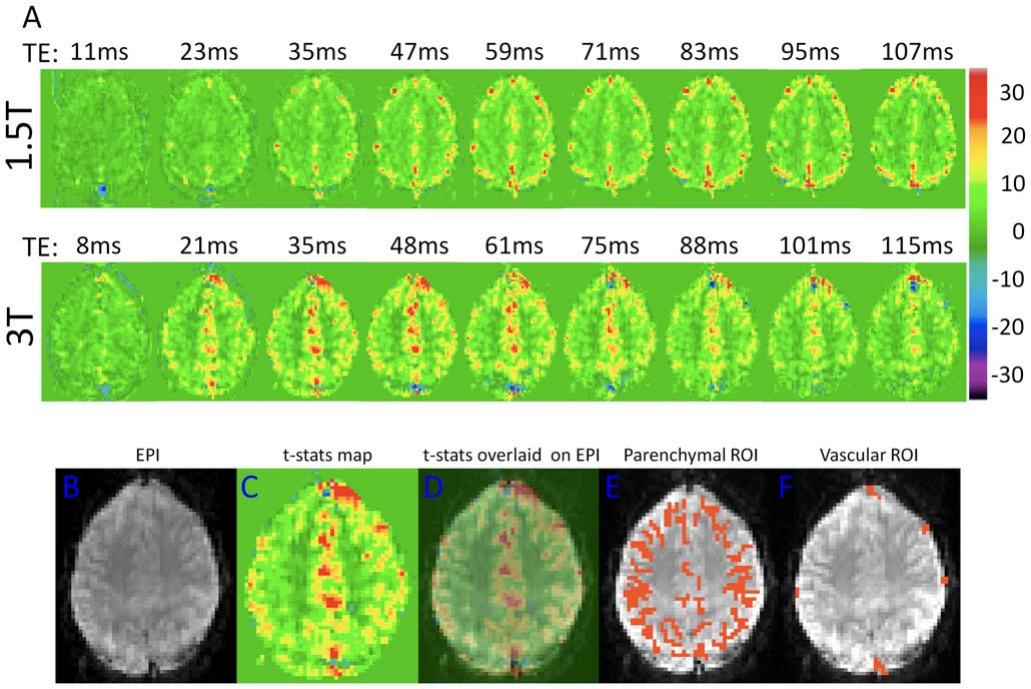
Activation maps from an individual subject. (A) Maps of t-statistics for BOLD response at 1.5T (top row) and 3T (middle row) for the various TEs (in ms) from an individual subject. (B) Representative slice of the original EPI data, (C) is the corresponding map of BOLD t-statistics, and (D) is the same map overlaid onto the original EPI data. Sample ROIs used for parenchymal and vascular measurements, shown in (E) and (F), respectively.

Figure 1A shows maps of BOLD t-statistics generated by fitting a linear signal model to the dynamic image time-series in a representative subject, illustrating the TE dependence of regional BOLD contrast during hypercapnia at 1.5T (top row) and 3T (middle row). Since the t-statistics computed as the ratio of the estimated effect size (also defined as “the contrast”) to the residual standard model error (corresponding loosely to the “noise” of the time-series), the values in these maps can be viewed as the contrast-to-noise ratio with an additional factor to correct the statistics for the degrees of freedom of the model fit (which is the same for all images). The shortest TE (11 ms) maps acquired at 1.5T show no significant signal changes other than a prominent negative response in the posterior sagittal sinus, likely arising from increased flow dephasing of blood spins in the sagittal sinus due to higher flow velocity during hypercapnia. The next TE (23 ms) shows almost exclusively large veins. Increased BOLD contrast in parenchymal gray matter is apparent at longer TEs. While the activated areas at later TEs appear to be distributed nearly uniformly throughout cortical gray matter at 1.5T, there is a noticeable emphasis toward the outer perimeter of the cortex, possibly corresponding to strong signals in pial vessels with intermediate T_2_^*^ values. In short, the large veins visible at short TE remain prominent out to the longest TEs at 1.5T, although at the later times they appear against a background of parenchymal response. At 3T, responses in large veins are visible even at the shortest TE of 8 ms. This is presumably due to the enhancement of intravascular susceptibility effects (shorter T_2_^*^) in veins at higher field. The maps acquired with TE of 21 ms and greater show parenchymal responses with the majority of the cortex activated and a rapid reduction in the prominence of the large veins at echo-times above 35 ms. At 3T, we also observed a prominent positive and for later TEs negative response in the frontal areas. The prominent negative signal changes represent complex flow dephasing effects in the high-velocity blood flowing through the sagittal sinus.

Figure 1B shows a representative slice of the original EPI time-series data from a single subject, 1C is the corresponding map of BOLD t-statistics generated by fitting a linear signal model to the dynamic image time-series and figure 1D is the BOLD t-statistics map overlaid on the original EPI data showing correspondence with anatomy. Figures 1E and 1F show sample ROIs used for parenchymal and vascular measurements, respectively. Voxels were classified as “parenchymal” on the basis of having no MRI visible evidence of macroscopic veins. In reality, such voxels must nonetheless contain a mixture of neural tissues, venules, and very small veins. The parenchymal BOLD signal must therefore include a mixture of intra and extra-vascular responses, with the emphasis more heavily on the intra-vascular component at 1.5 T.

**Figure 2:**
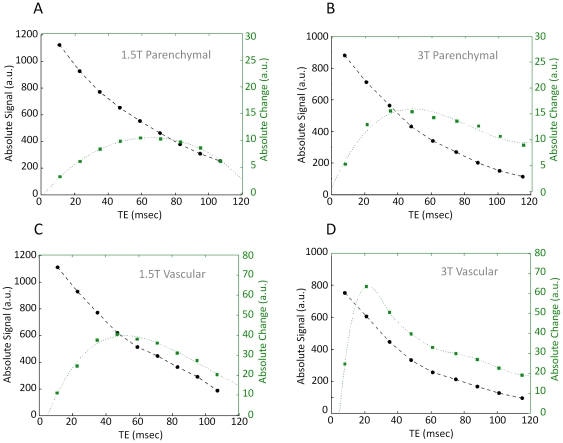
Absolute signal and absolute BOLD signal changes at 1.5T and 3T for parenchyma and veins. Dependence of the absolute signal and BOLD signal changes on TE at 1.5T (left) and 3T (right), for parenchymal (top row) and major veins (lower row). The absolute signal changes in the veins (blue graphs) attain the peak value at lower TE compared to the parenchymal at each field strength. Namely, the parenchymal response peaked at 62.0±11.5 ms and 41.5±7.5 ms for 1.5T and 3T respectively while the venous response peaked at 41.0±6.9 ms and 21.5±1.0 ms for the two field strengths. All measurements are averaged over all subjects and two scanning sessions at each field strength.

Figure 2 shows the average baseline MRI signal plots (arbitrary units, black circles) and the signal change during hypercapnia (arbitrary units, green squares) as a function of TE at 1.5T and 3T for gray matter (parenchymal) and large vein (vascular) ROIs. The ROI measures for these compartments were averaged over all subjects and scanning sessions. The plots of baseline signal show, in all cases, the expected quasi-exponential decay curve. Figure 2 also shows the signal change from hypercapnia as a function of TE. Fitting the signal change curves to the theoretical response given by,
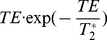
(8)provides an estimate of T_2_^*^ of 45.05 ms for vascular and 59.22 ms parenchymal tissue at 1.5T and 26.35 ms, 46.66 ms for vascular and parenchymal tissue at 3T respectively. Acceleration of signal decay in large veins is readily discernible in the data acquired at 3T, and it can be seen that the peak response amplitude for veins is noticeably shifted to shorter echo-times compared to the peak for the parenchymal response. There is considerably less TE separation between the peaks in the venous and parenchymal responses at 1.5T, consistent with the observation of strong responses for major veins against the parenchymal background at long echo-times in Figure 1.

**Figure 3:**
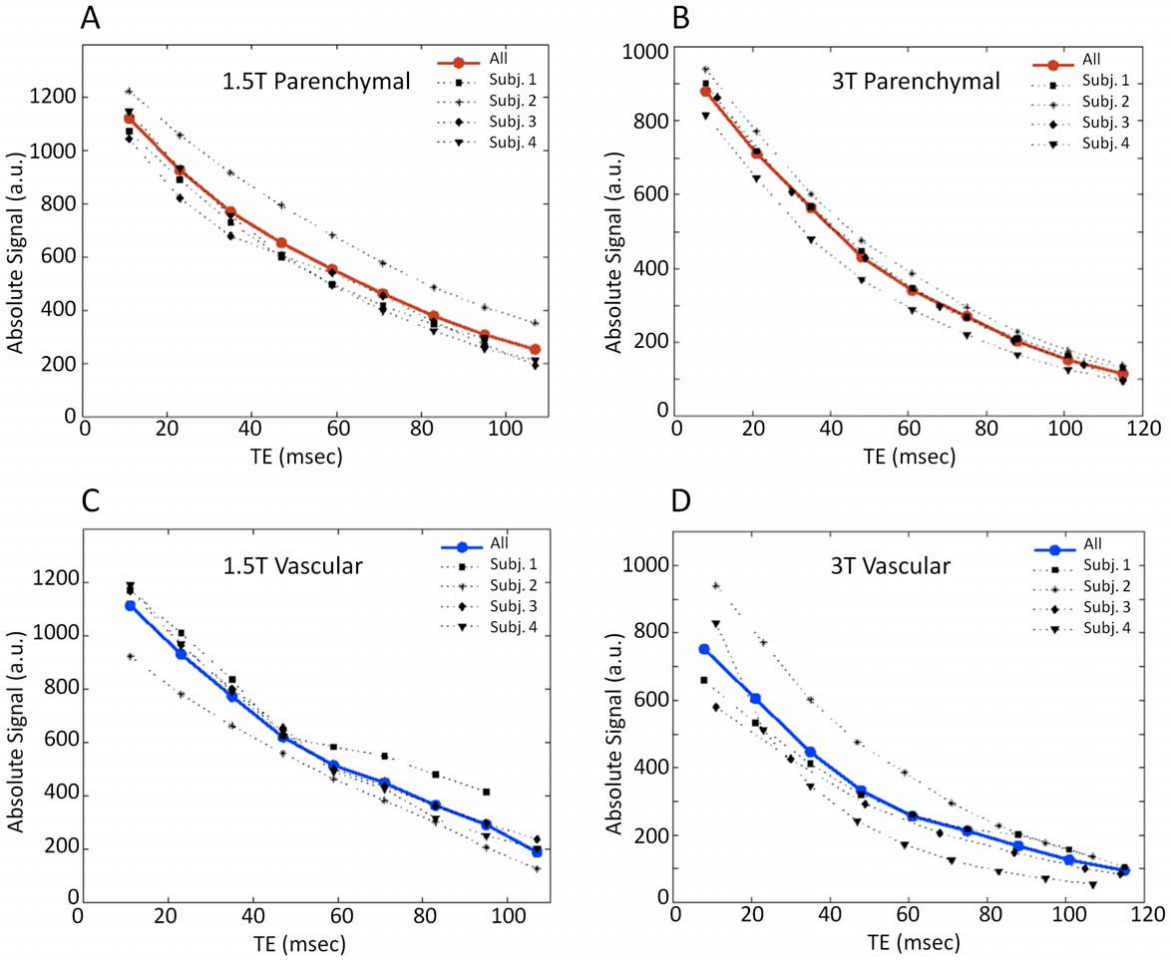
Absolute signal as a function of echo-time for individual subjects. Variations in dependence of the absolute signal on TE for the individual subjects at 1.5T (left) and 3T (right), in regions of parenchymal (A, B) and major veins (C, D). Red and blue circles with solid lines represent the average values over all subjects (as shown in Figure 2) for parenchymal and major veins, respectively. Illustrated data taken from two scanning sessions.

**Figure 4:**
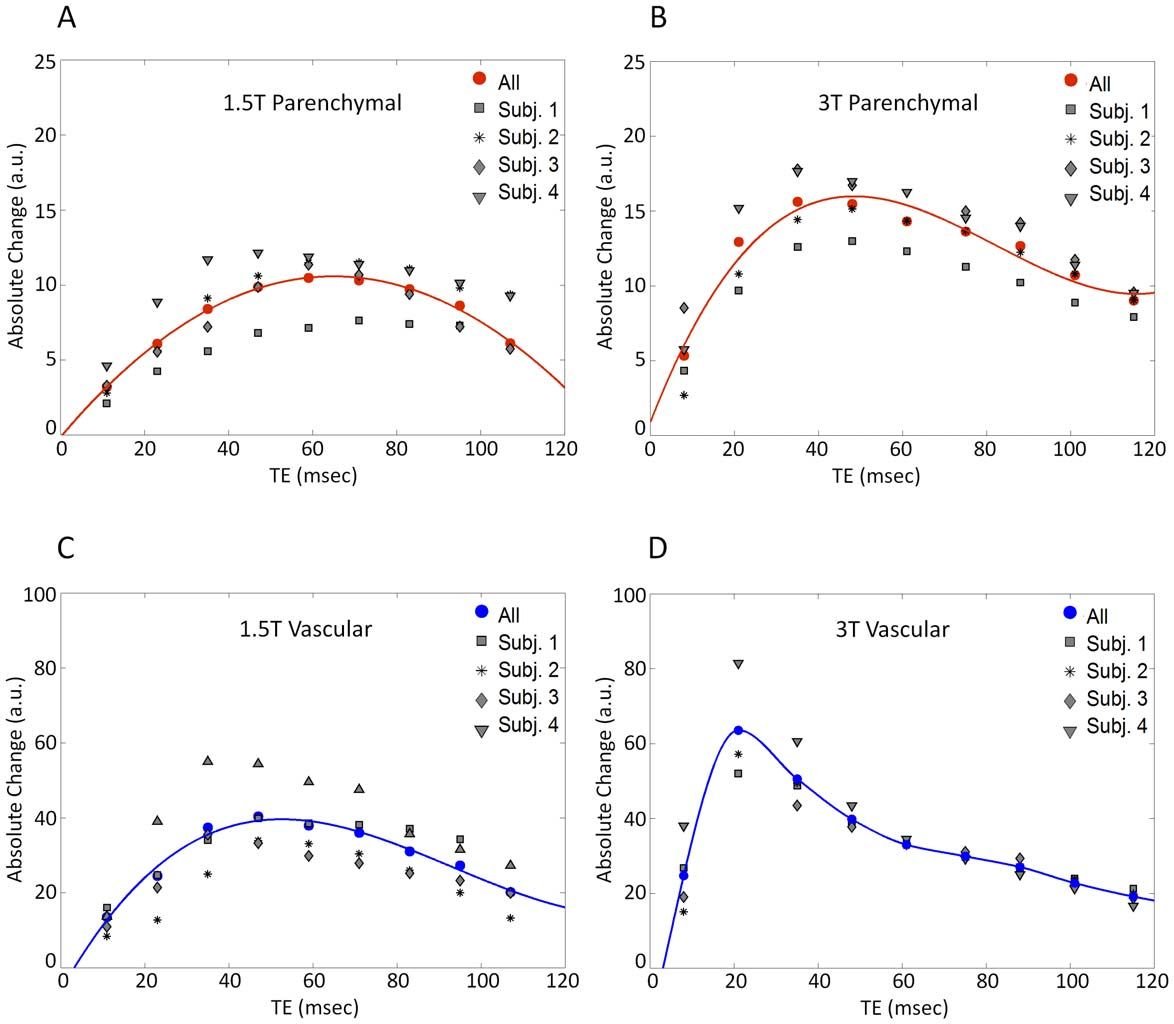
Absolute BOLD signal changes as a function of echo-time for individual subjects. Variations in absolute BOLD signal changes in parenchymal (A, B) and major veins (C, D) across TE for the individual subjects (gray symbols) at 1.5T (left) and 3T (right). Illustrated data taken from two scanning runs. Red and blue circles represent the average values over all subjects for parenchymal and major veins respectively. Measurements show that the absolute BOLD signal change on individual subjects peak at slightly different echo-times.

Table 1 presents the echo-times, which maximizes the BOLD signal change for different field strengths for all individual subjects (averaged over two scanning runs), as well as the mean values over all subjects. Peak absolute parenchymal responses were observed at echo-times of 62.0±11.5 ms and 41.5±7.5 ms at 1.5 and 3T respectively. The maximal venous responses were seen at echo-times of 41.0±6.9 ms and 21.5±1.0 ms at 1.5T and 3T, respectively, exhibiting a close correspondence to the theoretical estimates of T_2_^*^. Inter-subject variability of signal level as a function of TE is illustrated further in Figure 3 and Figure 4. Different subjects showed slightly different BOLD signal change dependence on the TE. The TE peaks were fairly broad, with a relatively flat maximal region covering an appreciable fraction of the total TE. The relatively large percent variability likely reflects a combination of the broadness of the TE peak, coupled with measurement variance and physiological effects, such as differences in hematocrit and baseline blood flow rates of the different individuals studied.

**Table 1:** Parenchymal and vascular echo-time (TE) peaks at 1.5T and 3T.

Subject	Peak TE (ms)1.5T Parenchymal	Peak TE (ms)3T Parenchymal	Peak TE (ms)1.5T Vascular	Peak TE (ms)3T Vascular
1	71	48	47	21
2	71	48	47	21
3	59	35	35	23
4	47	35	35	21
**Mean**	**62.0±11.5**	**41.5±7.5**	**41.0±6.9**	**21.5±1.0**

Peak TE at 1.5T and 3T for parenchyma and major veins for all individual subjects. Data are averaged over two scanning runs.

**Figure 5:**
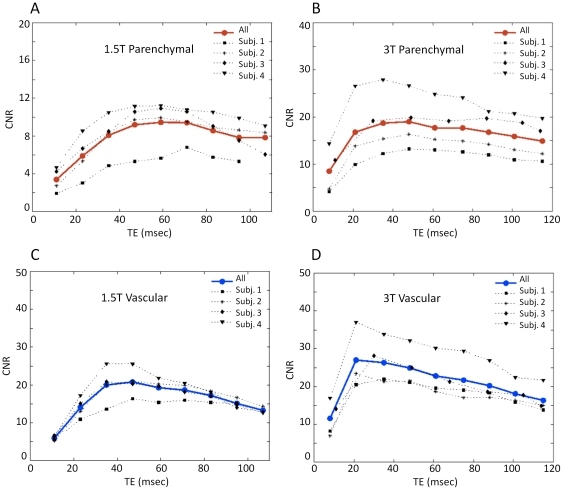
Contrast to noise ratio in parenchymal and major veins at 1.5T and 3T. CNR in parenchymal (top row) and major veins (bottom row) across TE for the individual subjects at 1.5T (left) and 3T (right). Illustrated data taken from two scanning runs. Red and blue circles represent the average values over all subjects for parenchymal and major veins respectively. Measurements showed that there is a slight inter-subject variability with the average over all subjects (red circles) peak at 65.00±6.93 ms for 1.5T and 44.8±6.5 ms for 3T. Similarly CNR of major veins has a small variation across subjects, with averages peaking at 41.0±6.9 ms for 1.5T and 26.8±6.9 ms for 3T.

Figure 5 illustrates inter-subject variability of the CNR in regions of parenchyma (red circles) and major veins (blue circles) as a function of TE at 1.5T and 3T. Peak parenchymal responses are obtained at different echo-times at the two field strengths. CNR at the major veins is shifted to shorter echo-times compared to the peak for the parenchymal response. The CNR at peak echo-times derived by the plot of t-statistics as a function of TE is shown in Figure 5. The parenchymal CNR at 3T was found to exceed that seen at 1.5T by a factor of 1.92±0.4.

**Figure 6:**
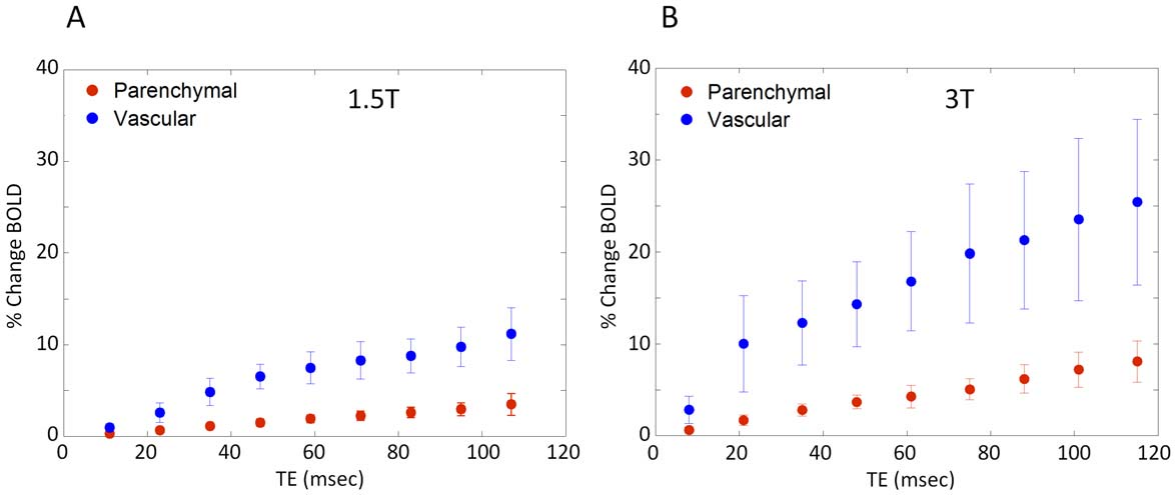
Percent BOLD signal change at 1.5T and 3T. Linear increase of the % activation induced BOLD signal change across TEs at 1.5T (A) and 3T (B). Parenchymal and vascular responses are shown in red and blue circles respectively showing steeper and larger responses as a function of TE at higher fields and for vascular compared to parenchymal.

**Figure 7:**
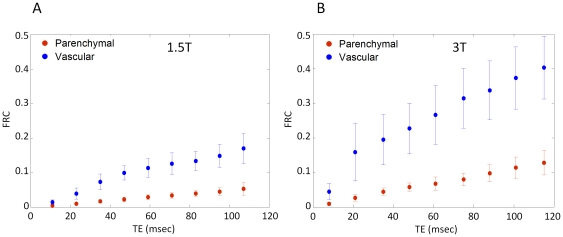
Flow Relaxation Coefficient (FRC) at parenchymal and vascular components. Flow relaxation coefficient, (%BOLD signal change normalized with the CBF measurements) at both 1.5T (A) and 3T (B) as a function of TE, for parenchymal (red) and vascular (blue) components. The flow changes were measured using ASL data acquired at the same session and the %BOLD signal changes are the data of Figure 6. The FRC at 3T is greater than that seen at 1.5T by a factor of 1.76±0.54 and 1.85±0.9 for parenchymal and vascular respectively measured at the TE of the peak BOLD signal change.

Figure 6 shows the percent BOLD signal change as a function of TE for the vascular and parenchymal ROIs at each field strength. The percent BOLD change increases roughly linearly with TE with a steeper slope for the vascular components compared to the parenchymal. For a given component the increase is steeper at the higher field. To control for possible differences in hypercapnic response amongst scanning sessions, acquisition protocols and different scanners, we calculated the FRC (%BOLD/%CBF increase). Figure 7 shows the FRC as a function of TE at 1.5T and 3T for both parenchymal and vascular components. The flow changes were measured using ASL data acquired at the same session. The FRC at 3T is greater than that seen at 1.5T by a factor of 1.76±0.54 and 1.85±0.9 for parenchymal and vascular respectively at the TE of the peak BOLD signal change. The FRC was 2.96 and 3.12 fold higher for veins than parenchyma at 1.5T and 3T respectively, showing a quantitative measure of the increase in specificity to parenchymal sources at 3T compared to 1.5T.

## Discussion

In this work, we investigated the effect of magnetic field strength and echo-time on the gradient-echo BOLD response in areas of cortical gray matter and resolvable veins during a global vasodilation. By calibrating the stimulus using direct CBF measurements and determining the BOLD response as a function of TE, we calculate the change in R_2_^*^ (ΔR_2_^*^) per unit fractional flow change (FRC) for each field strength at parenchymal and major veins.

Peak absolute parenchymal responses were observed at TE of 62.0±11.5 ms and 41.5±7.5 ms at 1.5 and 3T respectively. The maximal venous responses were seen at shorter echo-times; 41.0±6.9 ms and 21.5±1.0 ms at 1.5T and 3T, respectively. This suggests that to achieve maximum sensitivity and specificity, functional MRI experiments should be carried out using gradient-echo TE values at or above those at which peak *parenchymal* response was seen. This serves to avoid emphasis of venous response components, which can occur even at high field strength. Note that longer echo-times will also worsen susceptibility induced dephasing near air-filled sinuses. A survey of previous papers describing experiments at 1.5 and 3T [2], [22], [23] shows that the TE used in many studies are shorter than those recommended above to improve performance in regions near air-tissue interfaces. Our finding that shorter TE acquisitions weight the BOLD detection toward larger veins suggesting that this trade-off comes at a price in spatial localization. The average TE in the 1.5T studies surveyed was 43.3±5.8 ms, while the average for 3T studies was 35.0±7.1 ms (compare with 62.0±11.5 ms and 41.5±7.5 ms here). There has also been interest in extracting BOLD signals from short echo-time EPI data used in some ASL acquisitions in order to obtain simultaneous BOLD information in calibrated MRI schemes or other applications. Our data suggest that a multi-echo approach is preferable to avoid excessive BOLD bias toward large veins from the short echo-times often used in dedicated ASL scanning protocols.

The experiments described here suggest optimal echo-times for BOLD fMRI experiments performed at 1.5T and 3T under the specific conditions of our measurements. One condition, which, if varied, might lead to different results, is the spatial resolution of the EPI scans used. It can easily be shown, that the maximum BOLD signal change in a voxel is to be expected at TE equal to the T_2_^*^ value for that voxel. Since the decay process described by T_2_^*^ reflects intra-voxel dephasing that is related to the distribution of off-resonance frequency offsets within the voxel, larger voxels in a field gradient will have more frequency dispersion. Therefore we investigated whether the apparent T_2_^*^ value observed in parenchymal gray matter varied with voxel size in our EPI scans (data not shown). We tested this hypothesis by acquiring multi-echo scans at different spatial resolutions and fitting for T_2_^*^ at each voxel size. This exercise did not reveal any resolution-dependent changes in T_2_^*^ that would affect the TE results reported above, other than in close proximity to air-tissue interfaces. Regions close to such interfaces showed typical patterns of susceptibility dropout which, while less severe at higher spatial resolutions, would generally preclude reliable functional imaging.

To obtain a more general description of the relationship between the BOLD signal and perfusion changes at the two field strengths examined in this study, we also computed the fractional BOLD signal change per unit of fractional CBF change, the FRC, as a function of TE, at each field strength (Figure 7). From this, it was observed that BOLD signals at 3T exceeded those at 1.5T by a factor of 1.8 for parenchymal ROIs at TE corresponding to the peak %BOLD signal change. This knowledge is useful for interpreting quantitative differences seen at the two field strengths, or amongst different scanning sessions, but the more important predictor of sensitivity is the contrast-to-noise ratio. This comparison is provided by the plot of t-statistics as a function of TE (Figure 5), which showed peak parenchymal CNR values at TE of 65.0±6.9 ms and 44.8±6.5 ms at 1.5 and 3T respectively. Peak venous CNR was observed at respective TE of 41.0±6.9 ms and 26.8±6.9 ms for 1.5 and 3T, again shorter values than those for parenchyma. The parenchymal CNR at 3T was found to exceed that seen at 1.5T by a factor of 1.9±0.4 at the respective optimal TE. The FRC expresses the magnitude of the evoked BOLD response as a function of the underlying increase in CBF. This removes the impact of noise level, which can depend on factors such as coil performance, uncontrolled physiological fluctuations, sequence bandwidth, and voxel dimensions. While FRC and CNR are both important parameters, the FRC serves as a control to ensure that the field-dependent differences observed are not caused by systematic shifts in the efficiency of gas delivery on the two scanner platforms. The information conveyed in the FRC plots shown in Figure 7 is equivalent to a summary of parenchyma/vascular response ratios.

Note that the effect of underlying CBV changes during the hypercapnia experiments (Eq. 3 and 4) is a slight reduction in the amplitude of BOLD response compared with what would be observed if CBV were constant. Chen and Pike [24] using quantitative measurements of venous CBV, showed the venous volume responses in response to hypercapnia were comparable to those observed during neuronal activation. These findings support our results obtained using the hypercapnic manipulation are also relevant for activation studies.

Higher field strengths are generally accepted as having better specificity for parenchymal responses. This is consistent with the observations made in this study, but it should be noted that highly prominent responses at the major veins were still seen at 3T. A recent modeling study [5] described the fraction of the BOLD signal originating from the micro and macro vasculature at different field strengths and echo-times. Our data demonstrate micro and vascular effects which in general support the proposed model in [5], while illustrating the effects in actual activation maps showing responses to a global stimulus. The global nature of this stimulus makes it uniquely suited to the demonstration of sensitivity and specificity. However, it is possible that variations in major veins reactivity to these two types of event might lead to slightly different results. All measurements made in the present study were performed using gradient-echo EPI. As mentioned in the introduction, spin-echo techniques have attracted increasing interest at the highest field strengths in use at this time (e.g. 7T). However, given the broad availability of 1.5T and 3T MRI systems at clinical and research sites, we chose to focus on these field strengths and on the gradient-echo techniques that are most widely applied.

In conclusion, venous and parenchymal BOLD responses to a global challenge (hypercapnia) were investigated at field strengths of 1.5T and 3T. Peak responses in major veins were both stronger and occurred at shorter echo-times than parenchymal responses. At longer echo-times, the response was therefore more parenchymal weighted, especially for the 3T studies where the venous was seen to clearly diminish at the longest echo-times. Functional experiments should therefore be carried out at or around the TE at which peak parenchymal responses maximize sensitivity in order to maximize a combination of sensitivity and specificity. In other words longer TEs might be helpful in reducing contributions from microvasculature both at 1.5T and 3T. To ensure consistent percent BOLD change across multiple scanning sessions (either across scanners, inter-session variations, or in longitudinal studies), a new quantitative index was proposed, the FRC, defined as the fractional BOLD signal change per unit of fractional CBF change. The FRC ratios by tissue type should be closely related to the equivalent ratios of percent BOLD change if the manipulations (hypercapnia) were consistent, therefore it could be used as a control to ensure that the hypercapnic manipulations performed, for example at the two field strengths, produced equivalent CBF changes.
